# Egr3 Induces a Th17 Response by Promoting the Development of γδ T Cells

**DOI:** 10.1371/journal.pone.0087265

**Published:** 2014-01-24

**Authors:** Rose M. Parkinson, Samuel L. Collins, Maureen R. Horton, Jonathan D. Powell

**Affiliations:** 1 The Sidney-Kimmel Cancer Research Center, The Johns Hopkins School of Medicine, Baltimore, Maryland, United States of America; 2 Division of Pulmonary Medicine, Johns Hopkins University School of Medicine, Baltimore, Maryland, United States of America; Institut Pasteur, France

## Abstract

The transcription factor Early Growth Response 3 (Egr3) has been shown to play an important role in negatively regulating T cell activation and promoting T cell anergy in Th1 cells. However, its role in regulating other T helper subsets has yet to be described. We sought to determine the role of Egr3 in a Th17 response using transgenic mice that overexpress Egr3 in T cells (Egr3 TG). Splenocytes from Egr3 TG mice demonstrated more robust generation of Th17 cells even under non-Th17 skewing conditions. We found that while Egr3 TG T cells were not intrinsically more likely to become Th17 cells, the environment encountered by these cells was more conducive to Th17 development. Further analysis revealed a considerable increase in the number of γδ T cells in both the peripheral lymphoid organs and mucosal tissues of Egr3 TG mice, a cell type which normally accounts for only a small fraction of peripheral lymphocytes. Consistent with this marked increase in peripheral γδ T cells, thymocytes from Egr3 TG mice also appear biased toward γδ T cell development. Coculture of these Egr3-induced γδ T cells with wildtype CD4+ T cells increases Th17 differentiation, and Egr3 TG mice are more susceptible to bleomycin-induced lung inflammation. Overall our findings strengthen the role for Egr3 in promoting γδ T cell development and show that Egr3-induced γδ T cells are both functional and capable of altering the adaptive immune response in a Th17-biased manner. Our data also demonstrates that the role played by Egr3 in T cell activation and differentiation is more complex than previously thought.

## Introduction

Early growth response 3 (Egr3) is an immediate early zinc finger transcription factor activated in response to a variety of mitogenic signals [1]. Within the lymphocyte compartment, both mature and developing T cells highly express Egr3 shortly after TCR or pre-TCR engagement in a Ca^2+^ and NFAT-dependent manner [2], [3]. Our group has previously demonstrated a role for Egr3 in negative regulation of mature CD4 T cell function. *In vitro* anergized and self-antigen tolerized CD4+ T cells express significantly higher levels of Egr3 than cells fully activated in the presence of costimulation [4], [5]. Additionally, the overexpression of Egr3 in T cells promotes an anergic phenotype, inhibiting both proliferation and IL-2 production, while T cells lacking Egr3 are hyper-responsive to activation and fail to become tolerized in *in vivo* models of anergy [4], [5]. Egr3 negatively regulates T cell activation by inhibiting genes that induce IL-2 transcription [6] and by promoting transcription of FasL [7] and E3 ubiquitin ligases, which target proteins involved in TCR signaling for degradation [4], [8], [9]. Although its importance in T cell anergy has been established, these experiments were focused on Th1 effector cells. The influence of Egr3 on the development of other T helper subsets has not been previously investigated.

Th17 cells are critical in the defense against certain bacterial and fungal infections but also contribute to the pathogenesis of a number of autoimmune diseases. These cells produce the proinflammatory cytokines IL-17, IL-21, and IL-22 and their differentiation relies on the activation and expression of the transcription factors STAT3 and RORγt [10]–[12]. The precise mechanism by which Th17 T cells are produced *in vivo* remains controversial, but can involve IL-6 and TGFβ, IL-23 and IL-1, or some combination of these cytokines, with IL-23 believed to help maintain the Th17 phenotype or perhaps promote Th17 pathogenicity [13]–[16]. Many cell types can contribute to the Th17-biasing cytokine milieu, including nonlymphoid epithelial cells, innate immune cells like macrophages, DCs, and neutrophils, or even other activated T cells [17]–[19]. In addition to the downregulation of T cell activation machinery, Egr3 is thought to function in T cell development in part by inhibiting the function of RORγt in thymocytes [20], suggesting Egr3 might inhibit both Th1 and Th17 differentiation.

We sought to determine the role of Egr3 in the Th17 response using mice that transgenically overexpress Egr3 in T cells (Egr3 TG). Surprisingly, we found an increased proportion of CD4+ T cells from these mice produced IL-17 when stimulated under non-skewing conditions. This increase in Th17 cells does not appear to be a direct result of Egr3 overexpression, but instead correlates with an increase γδ T cell production in Egr3 TG mice. Our data highlight the ability of peripheral γδ T cells to markedly influence the adaptive helper T cell response. We show here that these Egr3-induced γδT cells function similarly to wildtype γδT cells, in that they produce Th17-polarizing cytokines, are capable of skewing CD4+ T cells to a Th17 phenotype, and can promote inflammatory disease in a model of pulmonary fibrosis. We also show that, contrary to findings in thymocyte populations, Egr3 expression does not inhibit Th17 activation in the periphery.

## Materials and Methods

### Mice

Egr3 TG mice on the B6.AKR background were obtained from G. Kersh, Emory University School of Medicine, Atlanta, Georgia [21]. The Egr3 TG mice were backcrossed onto C57 Bl/6 mice from Jackson Laboratories for at least five generations. This study was carried out in accordance with recommendations in the Guide for the Care and Use of Laboratory Animals of the National Institutes of Health. The protocol was approved by the Johns Hopkins University Institutional Animal Care and Use Committee.

### Cells, Abs, and reagents

Stimulatory anti-CD3 (2C11) and anti-CD28 (37.51) were isolated by affinity chromatography from hybridoma supernatants. Cytokines were purchased from Peprotech. PMA and ionomycin were purchased from Sigma. CD3-PE, CD4-APC, CD4-PE, CD8a-PE, CD8a-APC, CD8b-PE, CD8a-PerCP, γδTCR-FITC, (GL3), Vγ4-FITC (UC310A6), CD25-PE, CD44-FITC, CD44-PerCP Cy5.5, CD62L-APC, CD90.1-PE, IFNγ-APC, and IL-17-PerCP Cy5.5 antibodies were purchased from BD biosciences and used for flow cytometry. Vg1-FITC (2.11) and Vg7-FITC (F2.67) antibodies were generously donated by the Pereira lab. Lymphocytes were isolated from lung as described under the "Pulmonary Fibrosis Model." To isolate lymphocytes from small intestine and colon, tissues were harvested, sliced open longitudinally, washed several times in saline, and cut into small pieces. These pieces were then incubated with 0.1 mM EDTA, washed, then incubated in 1 mg/ml collagenase. Supernatant from these steps were collected, strained, and lymphocytes were separated from remaining epithelial cells using a 40% 60% Percoll gradient centrifugation step.CD4+ T cells were isolated from pooled spleen and lymph nodes of Egr3 TG mice using the CD4+ T cell negative isolation kit (Miltenyi Biotec). γδ T cells were isolated by enriching splenocytes using a T cell negative isolation kit (Miltenyi Biotec) and positively selecting for γδ T cells using a biotinylated γδ TCR Ab (BD biosciences), streptavidin coated beads, and magnetic columns (Miltenyi Biotec). CD4+ CD44- CD62L+ naive T cells were collected by FACS sorting. The Heilig and Tonegawa nomenclature is used when referring to specific subsets of γδ T cells.

### T Cell Activation

For activation of T cells with APC costimulation, 10^7^ whole splenocytes were cultured in a 6 well plate with soluble anti-CD3 at 2 µg/ml in 3 ml of media. After 48 h of stimulation, cells were collected, then added to 15 ml fresh media and cultured an additional 4 days. For CD4+ activation, 5×10^6^ purified CD4+ T cells or 5×10^6^ naive CD4+ T cells were stimulated in 1 ml in a 12 well dish with 2 µg/ml plate-bound anti-CD3 and 2 µg/ml soluble anti-CD28. After 48 hours, CD4+ T cells were expanded into 5 ml fresh media in a 6 well dish. All Th17 skewing was done by including IL-6 (10 ng/ml), TGFβ (5 ng/ml), and IL-23 (10 ng/ml) in the initial stimulation media. No exogenous cytokines were added to Th0 stimulation media or γδ coculture media. For γδ skewing experiments, purified γδ T cells were added to stimulation reaction at 1∶1 ratio with CD4+ T cells, keeping the total T cell number constant. All cells were restimulated on day 6 for 4-6 hours with PMA (50 ng/ml) and ionomycin (500 ng/ml) prior to intracellular cytokine staining. For RT-PCR of splenocytes, cells were collected and lysed prior to stimulation (day 0), 24 hours post-stimulation (day 1), or after 48hour of stimulation and 4 days of rest followed by a 6 hour restimulation with 1 µg/ml plate-bound anti-CD3 and 2 µg/ml soluble anti-CD28 (day 6). For RT-PCR of purified T cells, freshly isolated CD4+ T cells and γδ T cells were stimulated 16 hours with 1 µg/ml plate-bound anti-CD3 and 2 µg/ml soluble anti-CD28 prior to cells lysis for RT-PCR.

### Quantitative Real Time PCR

Total RNA was isolated from cultured T cells using TRIzol reagent (Invitrogen). Reverse transcriptase was performed on 1 µg of total RNA with the SuperScript III reverse transcriptase kit (Invitrogen). Real-time PCR was performed using ready-made primer/probe sets for Egr3, Id3, IL-17A, IL-17F, IL-6, IL-21, IL23, IL-23R, RORγt,18S endogenous control, and 2X PCR Master Mix (Applied Biosystems) on the ABI 7500 machine. Data were analyzed using SDS 1.5 software (Applied Biosystems). RNA abundance was calculated using the Δ*C*t method normalized to control 18S RNA and is plotted as 2^−Δ*C*t^ of isolated sample or time point divided by an unstimulated wildtype control (fold increase over baseline) unless otherwise indicated.

### Pulmonary Fibrosis Model

Mice were anesthetized using ketamine and xylazine, and 0.025 U of bleomycin was instilled intratrachealy (i.t.). Mice were then monitored for weight loss and survival. To assess lymphocyte infiltration into the lungs, bleomycin was administered as above and mice were killed with a lethal injection of sodium pentathol (Ampro Pharmaceutical, Arcadia, CA). Bronchoalveolar lavage (BAL) was performed by cannulating the trachea then instilling and retrieving 1 ml of sterile normal saline. The entire lavage volume was centrifuged, and the cell pellet was resuspended in RPMI 1640 supplemented with FBS and antibiotics. Following cardiac perfusion, lungs were harvested, chopped in a petri dish with a sterile razor blade, then lightly crushed and washed with RPMI over a 10um cell strainer. Recovered lung-infiltrating lymphocytes were stimulated 5 hours for intracellular cytokine staining.

## Results

### Increased Th17 differentiation in Egr3 TG mice is cell extrinsic

**Figure 1:**
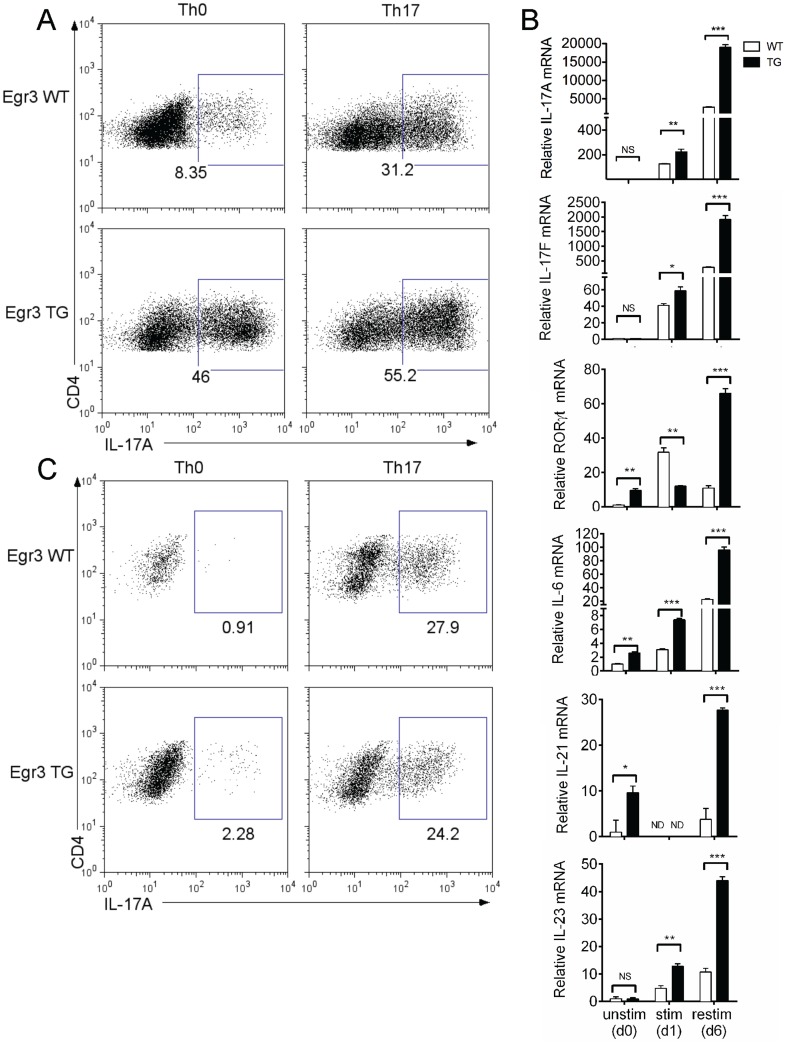
CD4+ T cells from Egr3 TG mice are biased toward Th17 differentiation. A-B) Splenocytes from 10-12 week old Egr3 WT and Egr3 TG mice were stimulated for 2 days with soluble anti-CD3 either with (Th17) or without (Th0) the addition of skewing cytokines. A) IL-17 production by Egr3 WT and Egr3 TG CD4+ T cells within the splenic population after restimulation. B) IL-17A, IL-17F, IL-21, RORγt, IL-23, and IL-6 mRNA expression in unstimulated (day 0), overnight stimulated (day 1) or previously activated and restimulated (day 6) splenocytes from Egr3 WT and Egr3 TG mice. Cells stimulated for mRNA were not skewed and mRNA expression was determined by qRT-PCR done in triplicate and normalized to 18s expression. C) Naive CD4+ T cells from Egr3 WT and Egr3 TG mice were stimulated with anti-CD3 and anti-CD28 for 2 days with or without skewing cytokines as in A, then restimulated and assessed for IL-17 production. Data are representative of 3 independent experiments, each using 2 mice per genotype. *p≤0.05, ** p≤0.01, ***p≤0.005

While the ability of Egr3 overexpression to inhibit T cell activation has been demonstrated [4], [5], this inhibition was measured through decreases in IL-2 production and proliferation, and the effects of Egr3 on T-helper differentiation have not been addressed. We hypothesized that Egr3 would inhibit Th17 differentiation in a similar fashion. To test this, we stimulated splenocytes from mice transgenically overexpressing Egr3 in T cells (Egr3 TG) or their wildtype counterparts (Egr3 WT) with or without the Th17 skewing cytokines IL-6, TGFβ, and IL-23. After two days of stimulation, cells were expanded into fresh media, rested, and then restimulated with PMA and Ionomycin on day 6 for intracellular cytokine staining. Both Egr3 WT and Egr3 TG CD4+ T cells produce IL-17 when splenocytes were stimulated under skewing conditions (Th17), and surprisingly, a large fraction of Egr3 TG CD4+ T cells also produced IL-17 under non-skewing (Th0) conditions (Figure 1a). To further explore this observation, we measured mRNA expression of various markers of the Th17 lineage by Egr3 WT and Egr3 TG splenocytes stimulated under non-skewing conditions. RORγt, IL-6 and IL-21 expression was higher in Egr3 TG splenocytes, even prior to stimulation (day 0). After only 24 hours of stimulation (day 1), IL-17A and IL-17 F expression was also higher in Egr3 TG splenocytes. However, the greatest differences between Egr3 WT and Egr3 TG splenocytes was found after splenocyte reactivation (day 6), when IL-17A, IL-17F, RORγt, IL-6, IL-21 and IL-23 mRNA levels were all significantly higher in Egr3 TG samples (Figure 1b). Such findings are consistent with the generation of Th17 cells and suggest that in the absence of exogenous skewing cytokines, the splenic environment in Egr3 TG mice is conducive to Th17 skewing. Many types of cells can contribute to the cytokine milieu in the spleen; therefore, to assess the intrinsic ability of Egr3 TG CD4+ T cells to produce IL-17, naive CD4+ T cells were sorted from Egr3 WT or TG splenocytes and activated in the absence (Th0) or presence (Th17) of skewing cytokines. Not surprisingly, isolated CD4+ T cells make less IL-17 than those stimulated in the presence of other splenocytes. More importantly, we also found that naive CD4+ T cells from both Egr3 WT and Egr3 TG mice have a similar capacity to become Th17 cells (Figure 1c). Though we have previously shown that Egr3 TG CD4+ T cells are hyporesponsive in their ability to proliferate and produce IL-2 [4], [5], our results here suggest that constitutive expression of Egr3 by T cells neither inhibits nor directly promotes Th17 generation in peripheral CD4+ lymphocytes.

### Egr3 promotes the development of γδ T cells

**Figure 2:**
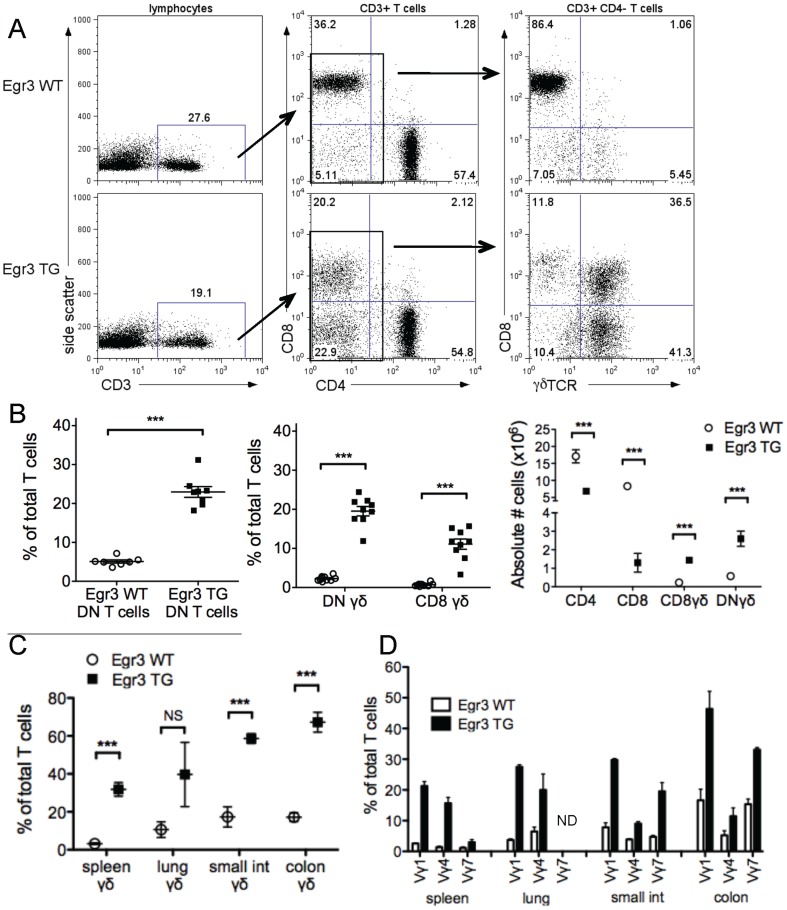
Increased number of γδ T cells in Egr3 TG mice. A-D) Splenocytes or mucosal lymphocytes harvested from untreated Egr3WT or Egr3TG mice were counted and stained for surface expression of CD3, CD4, CD8 and either γδTCR or a specific Vγ chain. A) CD3 expression shown as a fraction of total lymphocytes (left panels). Percentages of CD4+ T cells, CD8+ T cells, and CD4- CD8- T cells, shown as a fraction of CD3+ gated cells (center panels). γδTCR and CD8 expression shown as fraction of CD4- T cells (right panels). B) CD4- CD8- (DN) cells (left panel) or CD4- CD8- (DN) γδTCR+ and CD8+ γδTCR+ cells (center panel) shown as a percentage of total T cells (CD3+ gated splenocytes), with each dot representing a separate mouse. Averaged absolute numbers of T cell subsets per mouse spleen (right panel). Data in A and B are representative of 4 independent experiments, each using 2-3 mice per genotype. Absolute numbers in B are averaged from 4 experiments where splenocytes from 2-3 mice were pooled. C) γδ T cells found in the spleen, lung, small intestine, or colon of Egr3 WT or Egr3 TG mice, shown as a percentage of the CD3+ gated population. D) Percentage of cells expressing Vγ1, Vγ4, or Vγ7 chains, shown as a fraction of the CD3+ gated population. Data in C and D are representative of 3 independent experiments, each using lymphocytes pooled from 5-6 mice per genotype. ***p ≤ 0.005

Though Egr3 TG mice demonstrated an increase in splenic Th17 cells, the lymphoid organs of these mice contained a smaller proportion of total T cells (Figure 2a, left) as well as a reduced percentage of CD4+ and CD8+ T cells (Figure 2a, middle). This decrease in peripheral CD4+ and CD8+ T cells corresponds with a 4-8 fold increase in the percentage and absolute number of CD3+ T cells which lack expression of both CD4 or CD8 (Figure 2a, middle and Figure 2b), hereafter referred to as double negative T cells (DN) T cells. Upon further analysis, the majority of both the DN and CD8+ T cells from the spleens of Egr3 TG mice express a γδ TCR (Figure 2a, right). These DN γδ T cells and CD8+ γδ T cells together comprise nearly 35% of the T cell pool in Egr3 TG mice (Figure 2b, middle), a more than 5-fold increase in the number of γδ T cells (Figure 2b, right). Interestingly, the mean fluorescence intensity of CD8 is consistently half a log lower in Egr3 TG T cells (Figure 2a, middle), and closer inspection reveals that while very few CD8+ αβ T cells from either genotype express the CD8αα homodimer, there was a significantly higher proportion of CD8αα-expressing γδ T cells in Egr3 TG mice compared to their wildtype counterparts (Figure S1). The increase in γδ T cell numbers in these mice is not limited to the lymphoid organs, as similar increases were also detected in the lung and intestinal mucosa of Egr3 TG mice (Figure 2c). We subsequently analyzed the subsets of γδ T cells by their Vγ chain expression and found increases in all subsets that were assessed. The Vγ1 and Vγ4 subsets made up the largest fractions of γδ T cells in the lymphoid organs of Egr3 TG mice, while the majority of γδ T cells in the gut mucosa were either Vγ1 or Vγ7 subsets (Figure 2d).

The Egr transcription factors are known to play a role in thymocyte development and β-selection [21]–[24] and increasing evidence suggests that this transcription factor also plays a role in determining γδ T cells fate [25]–[27]. Developing γδ T cells express higher levels of Egr1, Egr2 and Egr3 as well as the transcriptional regulator Id3 [26], and ectopic expression of any of the Egrs can promote γδ TCR rearrangement *in vitro* [25], [27]. It has also been shown that mice that transgenically overexpress Egr1 have increased numbers of thymic γδ T cells. However, these studies did not address the functional properties of Egr1-induced γδ T cells in the periphery or the ability of Egr3 to promote development of γδ T cells that function in the periphery. Therefore, we assessed γδ T cell development in Egr3 TG mice in order to determine whether our mouse model aligned with these findings.

**Figure 3:**
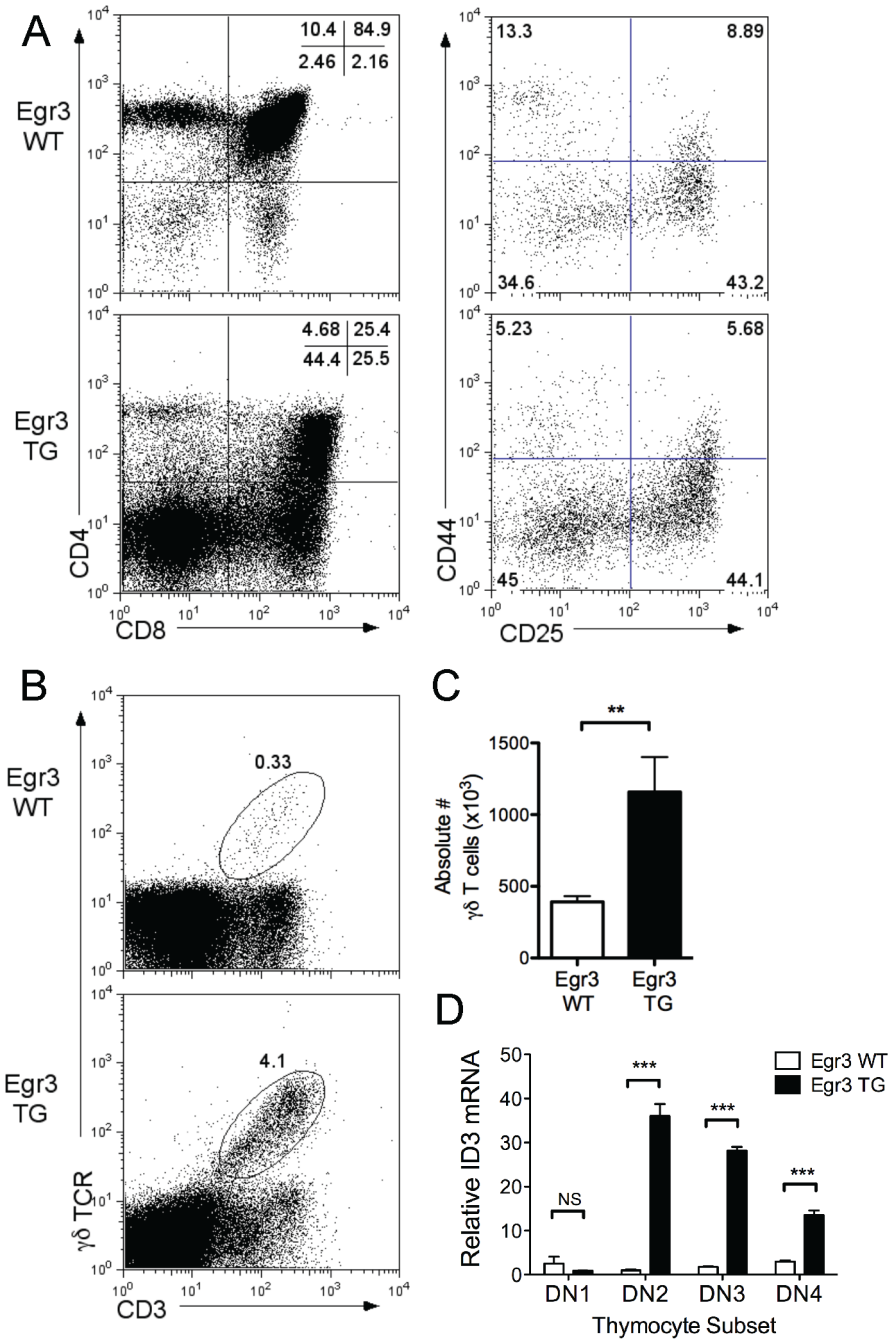
Thymocyte selection in Egr3 TG mice favors γδ T cells. A-B) Thymocytes harvested from littermate Egr3 WT and Egr3 TG mice were stained for surface marker expression or lysed for mRNA quantification. A) CD4 vs. CD8 expression was measured to determine double negative, double positive, and single positive subsets (left panels). DN1, DN2, DN3, and DN4 subsets shown as a percentage of the double negative fraction (right panels). B) γδT cells shown as a percentage of thymocytes (left panels) and C) γδT cells shown as an absolute number. D) Expression of Id3 mRNA in DN1, DN2, DN3, DN4 thymocytes assessed by qRT-PCR and normalized to 18s expression. Data in A-D are representative of 3 independent experiments, each using 2 mice per genotype. **p ≤ 0.01, ***p ≤ 0.005

To this end we harvested thymi from age-matched WT and Egr3 TG littermates and surface stained for the thymocyte subset markers CD4, CD8, CD44, and CD25. A decrease in thymic cellularity (not shown) and a robust increase in the percentage of CD4- CD8- (DN) thymocytes at the expense of the CD4+ CD8+ (DP) population in Egr3 TG mice (Figure 3a, left) is consistent with previous findings that Egr3 functions in survival and proliferation, during the DN to DP transition of developing αβ thymocytes [21], [22], [24]. Further investigation revealed a slight increase in the proportion of the CD44-, CD25- (DN4) thymocyte subset (Figure 3a, right) and an increase in both the percentage and absolute number of γδ T cells in the thymus (Figure 3b and 3c). It has been proposed that a strong TCR stimulus during thymic development, mediated by Id3, can promote γδ development over αβ selection [27]. To determine whether Egr3 might promote γδ development through modulation of Id3, we sorted freshly isolated CD4- CD8- thymocytes into DN1 through DN4 subsets and compared the mRNA expression of Id3 in Egr3 WT and Egr3 TG samples by qRT-PCR. Id3 expression was increased in the DN2 through DN4 subsets from Egr3 TG mice (Figure 3d). These results, along with the increased absolute number of peripheral γδ T cells in Egr3 TG mice (Figure 2b, right), support a role for Egr3 in promoting γδ T cell selection and inhibiting αβ T cell development in the thymus.

### γδ T cells from Egr3 TG mice are capable of skewing CD4+ T cells to Th17

**Figure 4:**
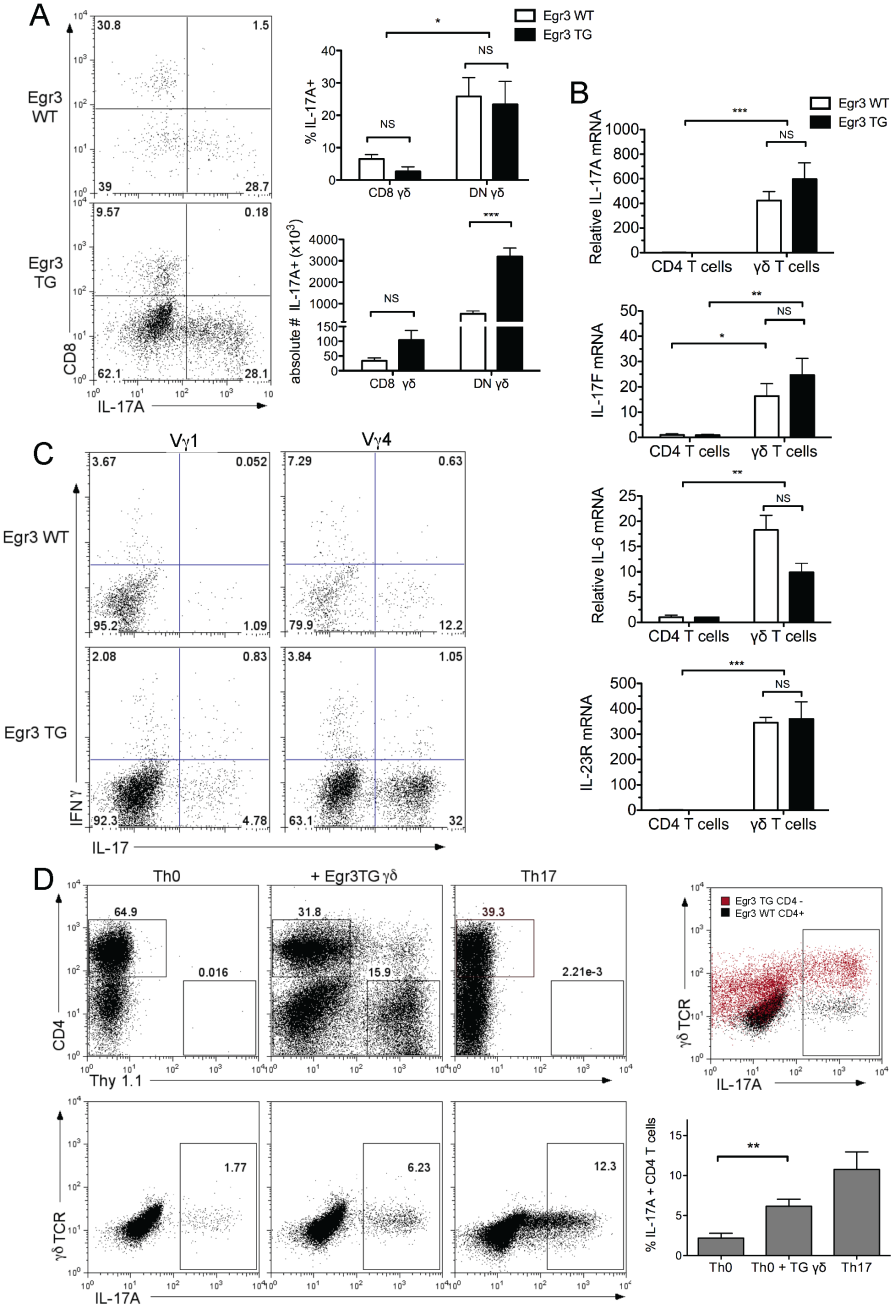
Egr3 TG γδ T cells produce IL-17 and skew CD4+ T cells to a Th17 phenotype. A) Splenocytes were stimulated and rested, and IL-17 production by CD8 γδ and DN γδ T cells was measured on day 6 after short restimulation (flow plots). The percentage of each subset producing IL-17 (top bar graph) and absolute number of IL-17 producing cells of each subset (bottom bar graph) are shown as an average of 4 independent experiments, each using 2 mice per genotype. B) Purified CD4+ T cells or γδ T cells were stimulated directly *ex vivo* with plate bound anti-CD3 and soluble anti-CD28 for 16 hours. IL-17A, IL-17F, IL-6, and IL-23R mRNA expression was measured by qRT-PCR done in triplicate and normalized to 18s expression. Data are representative of 3 independent experiments, each using 2 mice per genotype. C) IL-17 production measured as a percentage of either the Vγ1-expressing subset (left panels) or the Vγ4-expressing subset (right panels) of γδ T cells in splenocyte stimulation. Data are representative of 1 experiment using 5 mice per genotype. D) Egr3 WT CD4+ T cells were stimulated for 2 days with plate bound anti-CD3 and soluble anti-CD28 either alone (Th0), with the skewing cytokines TGFβ, IL-6, and IL-23 (Th17), or with equal numbers of Egr3 TG γδ T cells. IL-17 production by was assessed on day 6 after restimulation. CD4 and Thy1.1 expression shows the gating strategy used in this experiment (top row). IL-17 expression by the CD4+ Thy1.1- gated Egr3 WT T cells (bottom row). The overlay flow plot (top row, right) shows both the Egr3 WT CD4+ Thy1.1- T cells and Egr3 TG Thy1.1+ gates from the co-culture. Plots are representative of 3 independent experiments, which are averaged in the bar graph (bottom row, right). *p≤ 0.05, ** p≤ 0.01, ***p≤ 0.005

Having demonstrated a role for Egr3 in promoting the generation of γδ T cells and determining that these γδ T cells are capable of populating the peripheral lymphoid organs, we wanted to determine whether γδ T cells in the lymphoid compartment of Egr3 TG mice were functionally similar to those from Egr3 WT mice. When splenocytes from Egr3 WT and Egr3 TG littermate mice were activated, DN γδ T cells from both genotypes produced significantly more IL-17 than CD8+ γδ T cells, and the fraction of γδ T cells producing IL-17 was equivalent between genotypes (Figure 4a). Yet due to differences in their proportions within the spleen, there was a greater than 5-fold increase in absolute number of IL-17-producing γδ T cells in the Egr3 TG sample (Figure 4a, bottom bar graph). Early mRNA expression of IL-17A IL-17F, IL-6, and IL-23R was also found to be comparable between Egr3 WT and Egr3 TG γδ T cells (Figure 4b). It is thought that cytokine expression by γδ T cells may segregate in part according to different Vγ subsets, with Vγ4 T cells being a common producer of IL-17 [28]–[30]. To assess this in our model, we measured IL-17 production by the two γδ T cell subsets with the largest increase in Egr3 TG mice. Not surprisingly, Vγ4 T cells from both Egr3 WT and Egr3 TG mice produced more IL-17 than Vγ1 T cells (Figure 4c). Though IFNγ production by these γδ subsets appears lower than expected, we confirmed IFNγ staining using stimulated αβ T cells from the same Egr3 WT mice as a control (Figure S2).

Our findings that both CD4+ T cells and γδ T cells from Egr3 TG mice are capable of expressing IL-17 were surprising, given that Egr3 is thought to inhibit both RORγt expression and function in thymocyte and γδ T cell models [20], [31]. While we observed that constitutive expression of Egr3 did not reduce RORγt expression at either early or late time points, our splenic activation model shows that RORγt expression was briefly inhibited after 24 hours of stimulation (Figure 1b and Figure S3). Our data support a model whereby activation-induced levels of Egr3 inhibit RORγt function temporarily, and that this inhibition is relieved after Egr3 expression decreases. In fact, this model has already been proposed to explain the role of Egr3 in γδ T cell development [20]. Taken together, our findings suggest that Egr3-induced γδ T cells are similar to wildtype γδ T cells in their ability to produce cytokine. The clearest difference between these genotypes is the increase in the absolute number of γδ T cells (Figure 2b), particularly the Vγ4+ γδ T cells, a subset shown to produce IL-17 and other cytokines involved in Th17 skewing and stabilization (Figure 2d and Figure 4).

It has been suggested that γδ T cells have the ability to directly skew CD4+ T cells through the production of IL-17 [18]. Our findings suggest that the increased numbers of γδ T cells found in Egr3 TG mice are able to contribute to this skewing milieu, in part through the expression of both IL-17 and IL-6. To directly test whether Egr3-induced γδ T cells can skew CD4+ T cells, we isolated CD4+ T cells from Thy1.1- Egr3 WT mice and stimulated them alone, in the presence of Thy1.1+ Egr3TG γδ T cells, or with Th17-skewing conditions. Note that the “readout” cells in this assay are wildtype CD4+ T cells, thus eliminating a cell-intrinsic role for Egr3 in regulating Th17 production. CD4 and Thy1.1 were used to differentiate cell types within the co-culture, as it otherwise may have been difficult to separate γδ T cells from CD4 T cells based on post-activation TCR expression (Figure 4d, top row). Plotting these two gates together (Figure 4d, top right) shows both that this gating strategy was successful and that large numbers of Egr3 TG γδ T cells in this co-culture experiment produce IL-17. The addition of γδ T cells to the stimulation consistently increased production of IL-17 by CD4+ T cells more than 2-fold (Figure 4d, bottom row and bar graph). This co-culture demonstrates the influence of γδ T cells during CD4+ T cell activation and expansion, suggesting that prolonged exposure of CD4+ T cells to Egr3-induced γδ T cells may promote the strong Th17 skewing seen in Egr3TG mice.

### γδ T cells from Egr3 TG mice promote inflammation in a model of pulmonary fibrosis

**Figure 5:**
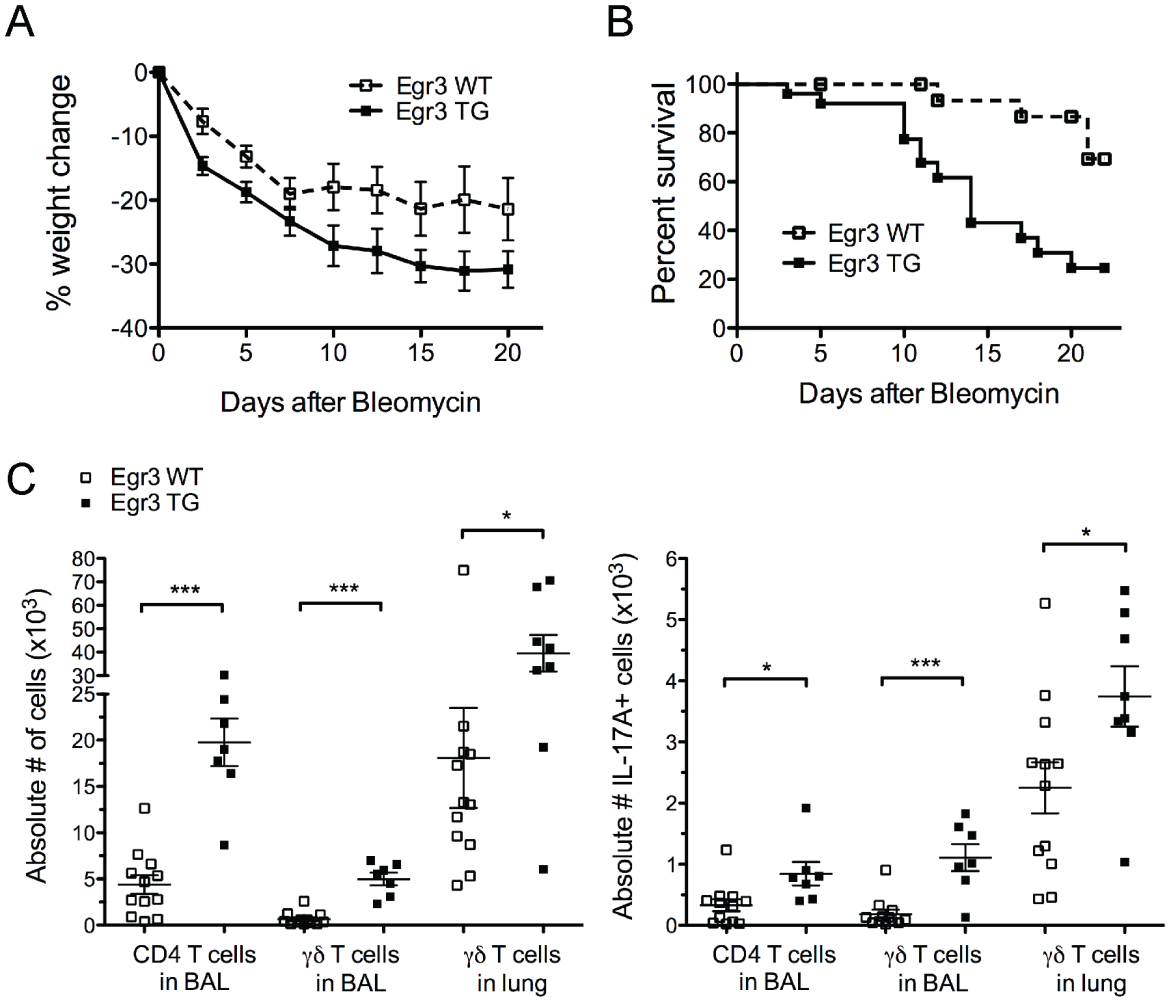
Egr3 TG mice are more susceptible in a model of pulmonary fibrosis. Egr3 WT or Egr3 TG mice were challenged with intratracheal bleomycin at day 0 and monitored for A) weight loss and B) survival. Data in A and B are compiled from 4 independent experiments. C) Separate mice were challenged as in A and sacrificed at day 5 to determine lymphocyte infiltration in the bronchaeolar lavage (BAL) and lung as well as the absolute number of IL-17-secreting cell infiltrates in each compartment. Data in C are representative of 3 independent experiments. *p≤ 0.05, ***p≤ 0.005

We next wanted to examine whether Egr3-induced γδ T cells could promote an *in vivo* inflammatory response. To this end, we utilized Egr3WT and Egr3TG mice in a model of pulmonary fibrosis, a disease in which both γδ T cells and IL-17 play a role [32], [33]. When mice were challenged with intratracheal (i.t.) bleomycin to induce inflammation and fibrosis, weight loss occurred faster and to a greater extent in Egr3TG mice compared to their Egr3 WT littermates (Figure 5a). In addition, a larger fraction of Egr3TG mice died prior to day 25 (Figure 5b). In light of these observations, we wanted to examine the inflammatory responses in the lungs of the Egr3WT and Egr3TG mice. Mice were given i.t. bleomycin and sacrificed 5 days later to determine the levels of cell infiltration and cytokine production within the lungs. Both the BAL and lungs of bleomycin-treated Egr3 TG mice contained 2-3 fold more total γδ T cells and a significantly higher number of IL-17-producing γδ T cells than in wildtype mice (Figure 5c). We also observed an increase in both total CD4+ T cell numbers and IL-17+ CD4+ T cell numbers in the BAL of Egr3 TG compared with Egr3 WT mice (Figure 5c). This finding is particularly striking considering Egr3 TG mice have impaired αβ T cell development and lower circulating numbers of CD4+ T cells (Figure 2). These data are consistent with the idea that Egr3-induced γδ T cells are capable of inducing inflammation and disease either directly or through their influence on CD4+ T cells.

## Discussion

Egr3 has been shown to be a negative regulator of CD4+ T cell responses and we initially undertook these experiments to examine the role of Egr3 in Th17 development. Surprisingly, we found that not only do Egr3 TG CD4+ T cells produce robust amounts of IL-17 but they skewed to become Th17 cells even under non skewing conditions. In pursuing this finding we revealed that this increase in Th17 differentiation in the Egr3 TG mice was not cell intrinsic. Rather, we demonstrate a role for Egr3 in the development of functional γδ T cells that can influence the adaptive immune response and promote inflammatory disease. These Egr3-induced γδ T cells reside in peripheral lymphoid organs as well as in circulation and in epithelial tissues, and are capable of producing cytokines at levels similar to wildtype γδ T cells. We found that though CD4+ T cells from Egr3 TG mice are similar to those from Egr3 WT mice in their capacity to secrete IL-17, they are skewed toward a Th17 fate, in part by the increased ratio of γδ T cells to CD4+ T cells in Egr3 TG mice. While we would have liked to compare the skewing ability of Egr3 WT and Egr3 TG γδ T cells in our co-culture experiment (Figure 4d), we were unable to isolate large enough numbers of Egr3 WT γδ T cell to do this. It is therefore not our intent to suggest that Egr3-induced γδ T cells are better at promoting a Th17 phenotype than wildtype γδ T cells. It has been shown that wildtype γδ T cells are capable of direct Th17 skewing [18], and our data highlight that Egr3-induced γδ T cells also possess this capability. Notably, our finding that Egr3-induced γδ T cells possess the functionality of wildtype γδ T cells, yet are present in larger numbers in the periphery, make them a potential model for further study of γδ T cells and their ability to influence the adaptive immune response.

While we demonstrate the ability of the γδ T cells to directly affect the skewing of CD4+ T cells, γδ T cells may also influence APCs and other cells in the lymphoid compartment to aid in Th17 skewing and maintenance. Our finding that IL-23 mRNA is elevated in Egr3 TG splenocytes (Figure 1b) supports this idea. This would also explain the greater difference in IL-17 production between genotypes in the presence of antigen presenting cells (APCs) and other splenocytes which could contribute to the skewing milieu (Figure 1a compared to Figure 4d). Our data support previous findings that innate-like γδ T cells can profoundly influence T helper cell differentiation and adaptive immune responses [18], [34], [35]. Our work also demonstrates an indirect role for Egr3 in promoting Th17 development, in which Egr3 promotes the development of γδ T cells which can in turn drive Th17 differentiation.

γδ T cells primarily produce either IFNγ or IL-17 and these populations can be segregated by Vγ chain usage or by surface marker expression. Vγ4+ cells are considered one of the most common IL-17 producers in the periphery [28], [30], and while IFNγ-producing γδ T cells are largely CD27+ NK1.1+, IL-17-producing γδ T cells are most often CD27– and CCR6+ [36], [37]. Though there were increases in all measured subsets of γδ T cells in Egr3 TG mice, one of the largest increases we found in the peripheral lymphoid organs was in this IL-17-associated Vγ4+ subset. This is not surprising, as we believe Egr3 influences γδ T cell development through promoting expression of Id3, and it has been reported that Id3-/- mice have a severe reduction of this particular subset of γδ T cells in the spleen [27]. Preliminary work by our group suggests that while NK1.1 and CCR6 expression is similar between Egr3 WT and Egr3 TG γδ T cells, IFNγ producing γδ T cells from Egr3 TG mice may be less likely to express CD27 than those from Egr3 WT mice (not shown). Future studies will more precisely define these findings.

It has also been suggested that IL-17 is produced by antigen-naïve γδ T cells, mediated by innate immune signals, while IFNγ production is induced by TCR stimuli [38]–[40]. The idea that TCR activation inhibits IL-17 production is supported by work showing that developing thymocytes which engage Skint-1 upregulate Egr3, which in turn suppresses RORγt expression and thus IL-17 production [31]. Though this would seem to conflict with our findings, most of the work by this group was done in thymocyte cultures, comparing Vγ5 and Vγ6 subsets of γδ T cells which normally populate the skin and reproductive mucosa. The *in vivo* skint-1 knock-out models show little to no increase in IL-17 production by peripheral γδ T cells [31], while our IL-17 producing Egr3-induced γδ T cells are both a different subset (Vγ4) and from peripheral lymphoid organs which lack Skint-1 expression.

Though the idea that γδ T cells can promote Th17 skewing is not new, we were initially surprised that CD4+ T cells and γδ T cells which overexpressed Egr3 were both capable of producing IL-17. Egr3 has been established as a mediator of T cell anergy, and its overexpression is thought to inhibit T cell activation [4], [5]. One possible explanation is that the level of Egr3 necessary for complete RORγt inhibition is not maintained in Egr3 TG mice. While Egr3 expression is higher in T cells from Egr3 TG mice both prior to- and at all time points after activation, the Egr3 transgene is expressed separately from the endogenous gene and the kinetics of Egr3 expression mimic that of the Egr3 WT mice, increasing to peak expression shortly after TCR engagement and returning to baseline levels just as quickly [21] (Figure S3). Egr3 has been shown to inhibit both RORγt expression and function during thymocyte development, though this inhibition is similarly transient; RORγt expression and function are restored once Egr3 expression drops [20]. With these latter points in mind, another possible explanation for increased IL-17 by Egr3-overexpressing CD4 T cells is that decreased IL-2 production by Egr3 TG T cells [5] balances any IL-17 inhibition by Egr3, as IL-2 plays a known role in inhibiting IL-17 expression[16], [41]. Overall, the finding that Egr3 TG CD4+ T cells are able to produce IL-17 has forced us to reconsider our definition of T cell anergy. Previous results led us to conclude that CD4+ T cells from Egr3 TG mice were anergic [4], [5], but a cell that proliferates more slowly and does not produce IL-2 or IFNγ is anergic only in the context of Th1 activation. We have shown here that transgenic overexpression of Egr3 does not inhibit Th17 differentiation in CD4+ T cells or IL-17 production by peripheral γδ T cells. While this is a negative finding, it shows that the role of Egr3 in IL-17 production is more complex than we originally believed.

It is widely accepted that the cytokine milieu experienced by a CD4+ T cell during primary activation is responsible for its differentiation into one of the many T helper subsets [42] and there is growing evidence to suggest that γδ T cells can contribute to this instructive environment [34], [35]. CD4+ T cells activated either in the presence of IL-6 and TGF-β, or IL-6, IL-1 and IL-23 both produce IL-17, but those skewed with TGF-β appear to be less inflammatory and fail to transfer susceptibility to experimental autoimmune encephalomyelitis (EAE) [15], [16]. Similarly, γδ T cells cultured *in vitro* with IL-23 and IL-1 produce IL-17 and have been linked to amplification of a Th17 response and exacerbation of inflammatory disease [18]. We have demonstrated here for the first time that Egr3 TG mice have an abundance of IL-17-producing γδ T cells, and that these Egr3-induced γδ T cells are capable of skewing CD4+ T cells *in vitro* without the addition of recombinant cytokines. Our data support not only the idea that γδ T cells shape the adaptive immune response, but also support a role for Egr3 in the development of this influential population of cells.

Though normally comprising 1-2% of circulating lymphocytes, γδ T cells are found more abundantly in epithelial and mucosal tissues. These cells are capable of responding rapidly to stress, tissue damage, and infection and can modulate the adaptive immune response in either a tolerogenic or inflammatory manner [43]–[48]. Recent work on the role of γδ T cells and IL-17 in pulmonary fibrosis suggests that γδ T cells amplify a Th17 response and promote inflammation, but differ on whether they amplify collagen deposition [32], help to control fibrosis via production of CXCL10 or IL-22 [32], [49]–[51] or have no effect on fibrotic disease [33]. In this report we demonstrate an increase in morbidity and mortality in Egr3 TG mice using a model of lung fibrosis. Previous work by our lab have shown that Egr3 TG mice are less susceptible to autoimmune pneumonitis [5], which at first seems to conflict with our current findings. However, while these experiments indicate less Th1 inflammation as measured by IFNγ production, total numbers lung infiltrates were similar and IL-17 was not measured. Along these lines, it remains to be determined if in the autoimmune pneumonitis model, the presence of increased Egr3 γδ T cells could actually be protective. Overall our findings suggest a role for Egr3 in promoting peripheral IL-17-producing γδ T cells, reveal a selective role for Egr3 γδ T cells in promoting tissue pathology, and offer a potential model for studying this population of cells.

## Supporting Information

Figure S1 **Higher percentage of Egr3 TG γδ T cells express CD8αα.** Egr3 WT and Egr3 TG splenocytes were gated on γδTCR+ T cells and γδTCR- T cells (αβ T cells) and expression of CD8α and CD8β chains were compared. Plots are representative of two experiments using 4 mice per genotype. Results are averaged in the bar graph.
(TIFF)

Figure S2 **IL-17 and IFNγ intracellular cytokine staining controls.** Egr3 WT splenocytes from the experiment shown in Figure 4c were gated on CD3+ γδTCR- cells (αβ T cells) and were either left unstimulated as a negative control (left panel) or stimulated as a positive control (right panel).
(TIFF)

Figure S3 **Egr3 TG T cells express higher levels of Egr3 and RORγt.** Purified γδ T cells were stimulated directly ex vivo for the times specified, then RORγt (A) or Egr3 (B) mRNA expression was measured by qRT-PCR done in triplicate and normalized to 18s expression. Data is from a single experiment using cells from 3 mice per genotype.
(TIFF)

## References

[1] Patwardhan S, Gashler A, Siegel MG, Chang LC, Joseph LJ, et al (1991) EGR3, a novel member of the Egr family of genes encoding immediate-early transcription factors. Oncogene 6: 917–928. 1906159

[2] Shao H, Kono DH, Chen LY, Rubin EM, Kaye J (1997) Induction of the early growth response (Egr) family of transcription factors during thymic selection. The Journal of experimental medicine 185: 731–744. 9034151 10.1084/jem.185.4.731 PMC2196139

[3] Mages HW, Stamminger T, Rilke O, Bravo R, Kroczek RA (1993) Expression of PILOT, a putative transcription factor, requires two signals and is cyclosporin A sensitive in T cells. International immunology 5: 63–70. 8443122 10.1093/intimm/5.1.63

[4] Safford M, Collins S, Lutz MA, Allen A, Huang C-T, et al (2005) Egr-2 and Egr-3 are negative regulators of T cell activation. Nature immunology 6: 472–480 10.1038/ni1193 15834410

[5] Collins S, Lutz MA, Zarek PE, Anders RA, Kersh GJ, et al (2008) Opposing regulation of T cell function by Egr-1/NAB2 and Egr-2/Egr-3. European journal of immunology 38: 528–536 10.1002/eji.200737157 18203138 PMC3598016

[6] Collins S, Wolfraim LA, Drake CG, Horton MR, Powell JD (2006) Cutting Edge: TCR-induced NAB2 enhances T cell function by coactivating IL-2 transcription. Journal of immunology (Baltimore, Md: 1950) 177: 8301–8305. 10.4049/jimmunol.177.12.8301 17142725

[7] Rengarajan J, Mittelstadt PR, Mages HW, Gerth AJ, Kroczek RA, et al (2000) Sequential Involvement of NFAT and Egr Transcription Factors in FasL Regulation. Immunity 12: 293–300 10.1016/S1074-7613(00)80182-X 10755616

[8] Heissmeyer V, Macián F, Im S-H, Varma R, Feske S, et al (2004) Calcineurin imposes T cell unresponsiveness through targeted proteolysis of signaling proteins. Nature immunology 5: 255–265 10.1038/ni1047 14973438

[9] Seroogy CM, Soares L, Ranheim EA, Su L, Holness C, et al (2004) The gene related to anergy in lymphocytes, an E3 ubiquitin ligase, is necessary for anergy induction in CD4 T cells. Journal of immunology (Baltimore, Md: 1950) 173: 79–85. 10.4049/jimmunol.173.1.79 15210761

[10] Huang Z, Xie H, Wang R, Sun Z (2007) Retinoid-related orphan receptor gamma t is a potential therapeutic target for controlling inflammatory autoimmunity. Expert opinion on therapeutic targets 11: 737–743 10.1517/14728222.11.6.737 17504012

[11] Harris TJ, Grosso JF, Yen H-R, Xin H, Kortylewski M, et al (2007) Cutting edge: An in vivo requirement for STAT3 signaling in TH17 development and TH17-dependent autoimmunity. Journal of immunology (Baltimore, Md: 1950) 179: 4313–4317. 10.4049/jimmunol.179.7.4313 17878325

[12] McGeachy MJ, Cua DJ (2008) Th17 cell differentiation: the long and winding road. Immunity 28: 445–453 10.1016/j.immuni.2008.03.001 18400187

[13] Veldhoen M, Hocking RJ, Atkins CJ, Locksley RM, Stockinger B (2006) TGFbeta in the context of an inflammatory cytokine milieu supports de novo differentiation of IL-17-producing T cells. Immunity 24: 179–189 10.1016/j.immuni.2006.01.001 16473830

[14] Zhou L, Ivanov II, Spolski R, Min R, Shenderov K, et al (2007) IL-6 programs T(H)-17 cell differentiation by promoting sequential engagement of the IL-21 and IL-23 pathways. Nature immunology 8: 967–974 10.1038/ni1488 17581537

[15] McGeachy MJ, Bak-Jensen KS, Chen Y, Tato CM, Blumenschein W, et al (2007) TGF-beta and IL-6 drive the production of IL-17 and IL-10 by T cells and restrain T(H)-17 cell-mediated pathology. Nature immunology 8: 1390–1397 10.1038/ni1539 17994024

[16] Ghoreschi K, Laurence A, Yang X-P, Tato CM, McGeachy MJ, et al (2010) Generation of pathogenic T(H)17 cells in the absence of TGF-β signalling. Nature 467: 967–971 10.1038/nature09447 20962846 PMC3108066

[17] Siegemund S, Schütze N, Schulz S, Wolk K, Nasilowska K, et al (2009) Differential IL-23 requirement for IL-22 and IL-17A production during innate immunity against Salmonella enterica serovar Enteritidis. International immunology 21: 555–565 10.1093/intimm/dxp025 19297659

[18] Sutton CE, Lalor SJ, Sweeney CM, Brereton CF, Lavelle EC, et al (2009) Interleukin-1 and IL-23 induce innate IL-17 production from gammadelta T cells, amplifying Th17 responses and autoimmunity. Immunity 31: 331–341 10.1016/j.immuni.2009.08.001 19682929

[19] Cua DJ, Tato CM (2010) Innate IL-17-producing cells: the sentinels of the immune system. Nature reviews Immunology 10: 479–489 10.1038/nri2800 20559326

[20] Xi H, Schwartz R, Engel I, Murre C, Kersh GJ (2006) Interplay between RORgammat, Egr3, and E proteins controls proliferation in response to pre-TCR signals. Immunity 24: 813–826 10.1016/j.immuni.2006.03.023 16782036

[21] Xi H, Kersh GJ (2004) Early growth response gene 3 regulates thymocyte proliferation during the transition from CD4-CD8- to CD4+CD8+. Journal of immunology (Baltimore, Md: 1950) 172: 964–971. 10.4049/jimmunol.172.2.964 14707069

[22] Xi H, Kersh GJ (2004) Sustained early growth response gene 3 expression inhibits the survival of CD4/CD8 double-positive thymocytes. Journal of immunology (Baltimore, Md: 1950) 173: 340–348. 10.4049/jimmunol.173.1.340 15210792

[23] Carter JH, Lefebvre JM, Wiest DL, Tourtellotte WG (2007) Redundant role for early growth response transcriptional regulators in thymocyte differentiation and survival. Journal of immunology (Baltimore, Md: 1950) 178: 6796–6805. 10.4049/jimmunol.178.11.6796 17513727

[24] Carleton M, Haks MC, Smeele SAA, Jones A, Belkowski SM, et al (2002) Early growth response transcription factors are required for development of CD4(-)CD8(-) thymocytes to the CD4(+)CD8(+) stage. Journal of immunology (Baltimore, Md: 1950) 168: 1649–1658. 10.4049/jimmunol.168.4.1649 11823493

[25] Haks MC, Lefebvre JM, Lauritsen JPH, Carleton M, Rhodes M, et al (2005) Attenuation of gammadeltaTCR signaling efficiently diverts thymocytes to the alphabeta lineage. Immunity 22: 595–606 10.1016/j.immuni.2005.04.003 15894277

[26] Taghon T, Yui MA, Pant R, Diamond RA, Rothenberg EV (2006) Developmental and molecular characterization of emerging beta- and gammadelta-selected pre-T cells in the adult mouse thymus. Immunity 24: 53–64 10.1016/j.immuni.2005.11.012 16413923

[27] Lauritsen JPH, Wong GW, Lee S-Y, Lefebvre JM, Ciofani M, et al (2009) Marked induction of the helix-loop-helix protein Id3 promotes the gammadelta T cell fate and renders their functional maturation Notch independent. Immunity 31: 565–575 10.1016/j.immuni.2009.07.010 19833086 PMC2768560

[28] Roark CL, French JD, Taylor MA, Bendele AM, Born WK, et al (2007) Exacerbation of collagen-induced arthritis by oligoclonal, IL-17-producing gamma delta T cells. Journal of immunology (Baltimore, Md: 1950) 179: 5576–5583. 10.4049/jimmunol.179.8.5576 PMC2768546 17911645

[29] O’Brien RL, Lahn M, Born WK, Huber SA (2001) T cell receptor and function cosegregate in gamma-delta T cell subsets. Chemical immunology 79: 1–28. 11478152 10.1159/000058829

[30] Narayan K, Sylvia KE, Malhotra N, Yin CC, Martens G, et al (2012) Intrathymic programming of effector fates in three molecularly distinct γδ T cell subtypes. Nature immunology 13: 511–518 10.1038/ni.2247 22473038 PMC3427768

[31] Turchinovich G, Hayday AC (2011) Skint-1 Identifies a Common Molecular Mechanism for the Development of Interferon-γ-Secreting versus Interleukin-17-Secreting γδ T Cells. Immunity 35: 59–68 10.1016/j.immuni.2011.04.018 21737317

[32] Wilson MS, Madala SK, Ramalingam TR, Gochuico BR, Rosas IO, et al (2010) Bleomycin and IL-1beta-mediated pulmonary fibrosis is IL-17A dependent. The Journal of experimental medicine 207: 535–552 10.1084/jem.20092121 20176803 PMC2839145

[33] Lo Re S, Dumoutier L, Couillin I, Van Vyve C, Yakoub Y, et al (2010) IL-17A-producing gammadelta T and Th17 lymphocytes mediate lung inflammation but not fibrosis in experimental silicosis. Journal of immunology (Baltimore, Md: 1950) 184: 6367–6377 10.4049/jimmunol.0900459 20421647

[34] Wang T, Gao Y, Scully E, Davis CT, Anderson JF, et al (2006) Gamma delta T cells facilitate adaptive immunity against West Nile virus infection in mice. Journal of immunology (Baltimore, Md: 1950) 177: 1825–1832. 10.4049/jimmunol.177.3.1825 16849493

[35] Ma Y, Aymeric L, Locher C, Mattarollo SR, Delahaye NF, et al (2011) Contribution of IL-17-producing gamma delta T cells to the efficacy of anticancer chemotherapy. The Journal of experimental medicine 208: 491–503 10.1084/jem.20100269 21383056 PMC3058575

[36] Ribot JC, deBarros A, Pang DJ, Neves JF, Peperzak V, et al (2009) CD27 is a thymic determinant of the balance between interferon-gamma- and interleukin 17-producing gammadelta T cell subsets. Nature immunology 10: 427–436 10.1038/ni.1717 19270712 PMC4167721

[37] Haas JD, González FHM, Schmitz S, Chennupati V, Föhse L, et al (2009) CCR6 and NK1.1 distinguish between IL-17A and IFN-gamma-producing gammadelta effector T cells. European journal of immunology 39: 3488–3497 10.1002/eji.200939922 19830744

[38] Jensen KDC, Su X, Shin S, Li L, Youssef S, et al (2008) Thymic selection determines gammadelta T cell effector fate: antigen-naive cells make interleukin-17 and antigen-experienced cells make interferon gamma. Immunity 29: 90–100 10.1016/j.immuni.2008.04.022 18585064 PMC2601709

[39] Martin B, Hirota K, Cua DJ, Stockinger B, Veldhoen M (2009) Interleukin-17-producing gammadelta T cells selectively expand in response to pathogen products and environmental signals. Immunity 31: 321–330 10.1016/j.immuni.2009.06.020 19682928

[40] Ribot JC, Chaves-Ferreira M, d’Orey F, Wencker M, Gonçalves-Sousa N, et al (2010) Cutting edge: adaptive versus innate receptor signals selectively control the pool sizes of murine IFN-γ- or IL-17-producing γδ T cells upon infection. Journal of immunology (Baltimore, Md: 1950) 185: 6421–6425 10.4049/jimmunol.1002283 PMC3915338 21037088

[41] Yang X-P, Ghoreschi K, Steward-Tharp SM, Rodriguez-Canales J, Zhu J, et al (2011) Opposing regulation of the locus encoding IL-17 through direct, reciprocal actions of STAT3 and STAT5. Nature immunology 12: 247–254 10.1038/ni.1995 21278738 PMC3182404

[42] Zhu J, Paul WE (2010) Peripheral CD4+ T-cell differentiation regulated by networks of cytokines and transcription factors. Immunological reviews 238: 247–262 10.1111/j.1600-065X.2010.00951.x 20969597 PMC2975272

[43] Jameson J, Ugarte K, Chen N, Yachi P, Fuchs E, et al (2002) A role for skin gammadelta T cells in wound repair. Science (New York, NY) 296: 747–749 10.1126/science.1069639 11976459

[44] Roberts SJ, Smith AL, West AB, Wen L, Findly RC, et al (1996) T-cell alpha beta + and gamma delta + deficient mice display abnormal but distinct phenotypes toward a natural, widespread infection of the intestinal epithelium. Proceedings of the National Academy of Sciences of the United States of America 93: 11774–11779. 8876213 10.1073/pnas.93.21.11774 PMC38134

[45] Han G, Wang R, Chen G, Wang J, Xu R, et al (2010) Interleukin-17-producing gammadelta+ T cells protect NOD mice from type 1 diabetes through a mechanism involving transforming growth factor-beta. Immunology 129: 197–206 10.1111/j.1365-2567.2009.03166.x 19824917 PMC2814462

[46] Cai Y, Shen X, Ding C, Qi C, Li K, et al (2011) Pivotal Role of Dermal IL-17-Producing γδ T Cells in Skin Inflammation. Immunity 35: 596–610 10.1016/j.immuni.2011.08.001 21982596 PMC3205267

[47] Petermann F, Rothhammer V, Claussen MC, Haas JD, Blanco LR, et al (2010) γδ T cells enhance autoimmunity by restraining regulatory T cell responses via an interleukin-23-dependent mechanism. Immunity 33: 351–363 10.1016/j.immuni.2010.08.013 20832339 PMC3008772

[48] Do J, Visperas A, Dong C, Baldwin WM, Min B (2011) Cutting edge: Generation of colitogenic Th17 CD4 T cells is enhanced by IL-17+ γδ T cells. Journal of immunology (Baltimore, Md: 1950) 186: 4546–4550 10.4049/jimmunol.1004021 PMC3200541 21402889

[49] Braun RK, Ferrick C, Neubauer P, Sjoding M, Sterner-Kock A, et al (2008) IL-17 producing gammadelta T cells are required for a controlled inflammatory response after bleomycin-induced lung injury. Inflammation 31: 167–179 10.1007/s10753-008-9062-6 18338242

[50] Simonian PL, Wehrmann F, Roark CL, Born WK, O’Brien RL, et al (2010) γδ T cells protect against lung fibrosis via IL-22. The Journal of experimental medicine 207: 2239–2253 10.1084/jem.20100061 20855496 PMC2947077

[51] Pociask DA, Chen K, Choi SM, Oury TD, Steele C, et al (2011) γδ T cells attenuate bleomycin-induced fibrosis through the production of CXCL10. The American journal of pathology 178: 1167–1176 10.1016/j.ajpath.2010.11.055 21356368 PMC3070585

