# Supplementary figures and images for: Egr3 Induces a Th17 Response by Promoting the Development of γδ T Cells
Source: PLoS One. 2014 Jan 24;9(1):e87265. doi: 10.1371/journal.pone.0087265 (PMC3901773; doi:10.1371/journal.pone.0087265)

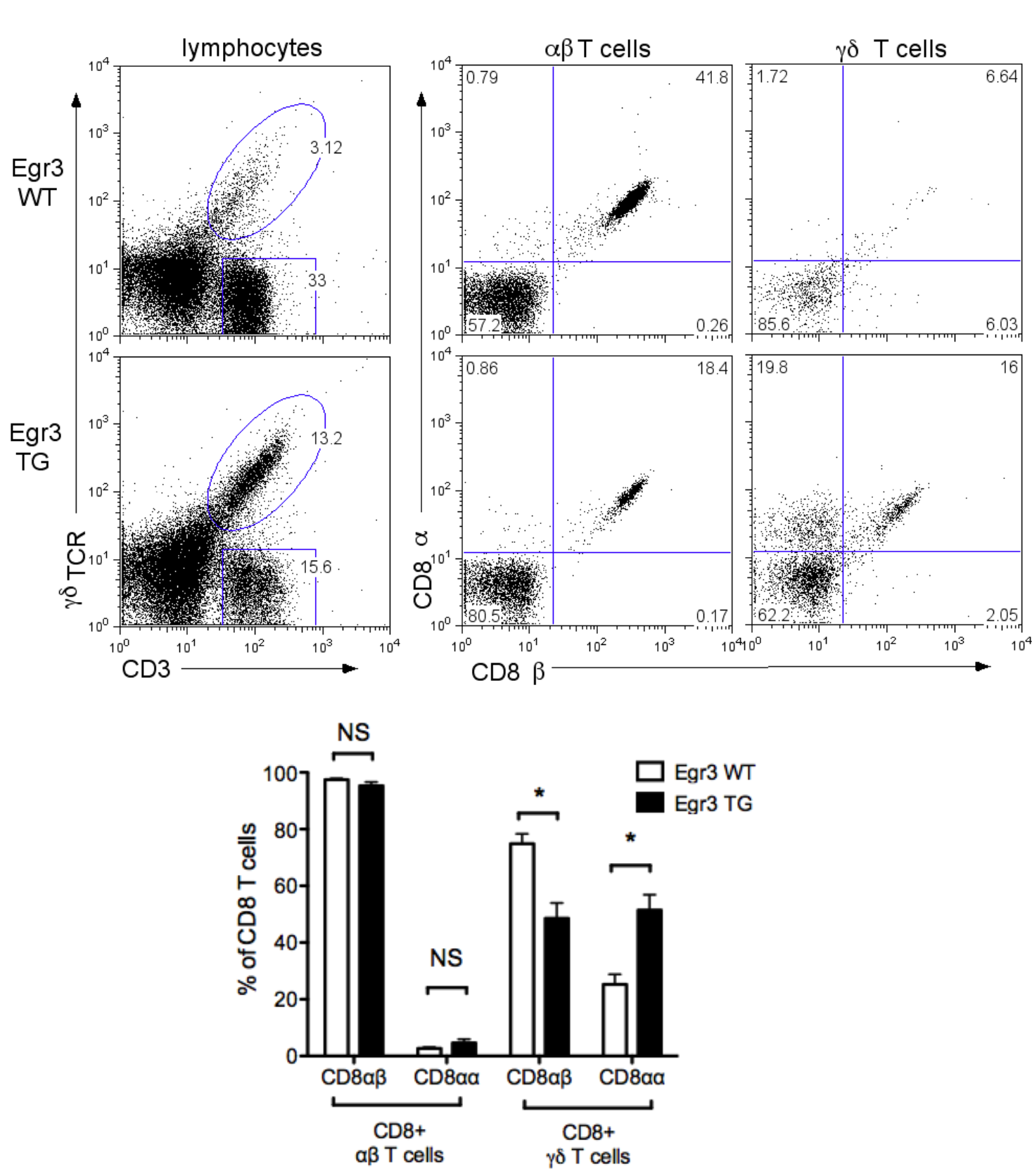

Supplement: Figure S1 — Higher percentage of Egr3 TG γδ T cells express CD8αα. Egr3 WT and Egr3 TG splenocytes were gated on γδTCR+ T cells and γδTCR- T cells (αβ T cells) and expression of CD8α and CD8β chains were compared. Plots are representative of two experiments using 4 mice per genotype. Results are averaged in the bar graph. (TIFF) [file pone.0087265.s001.tiff]

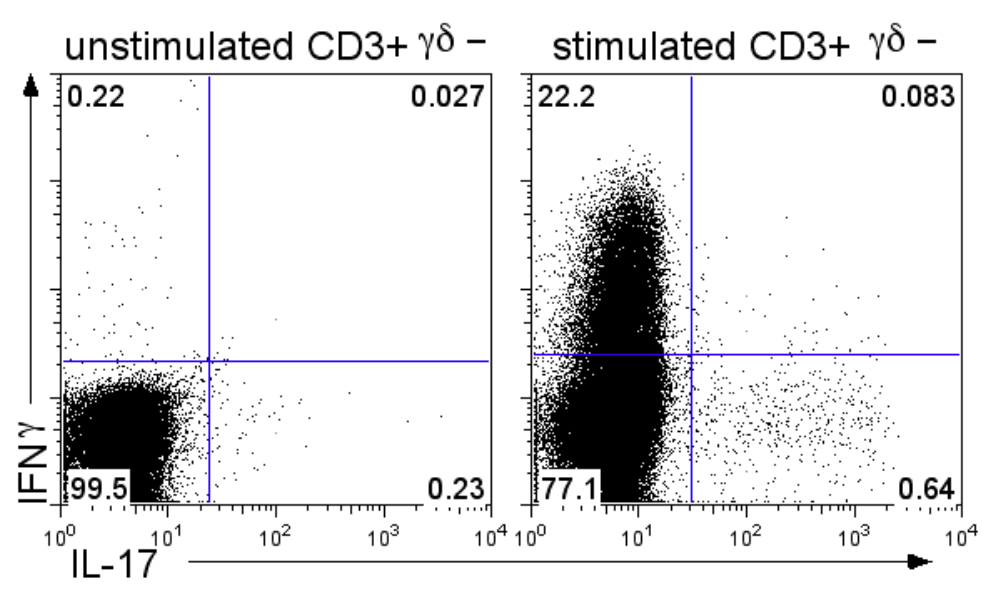

Supplement: Figure S2 — IL-17 and IFNγ intracellular cytokine staining controls. Egr3 WT splenocytes from the experiment shown in Figure 4c were gated on CD3+ γδTCR- cells (αβ T cells) and were either left unstimulated as a negative control (left panel) or stimulated as a positive control (right panel). (TIFF) [file pone.0087265.s002.tiff]

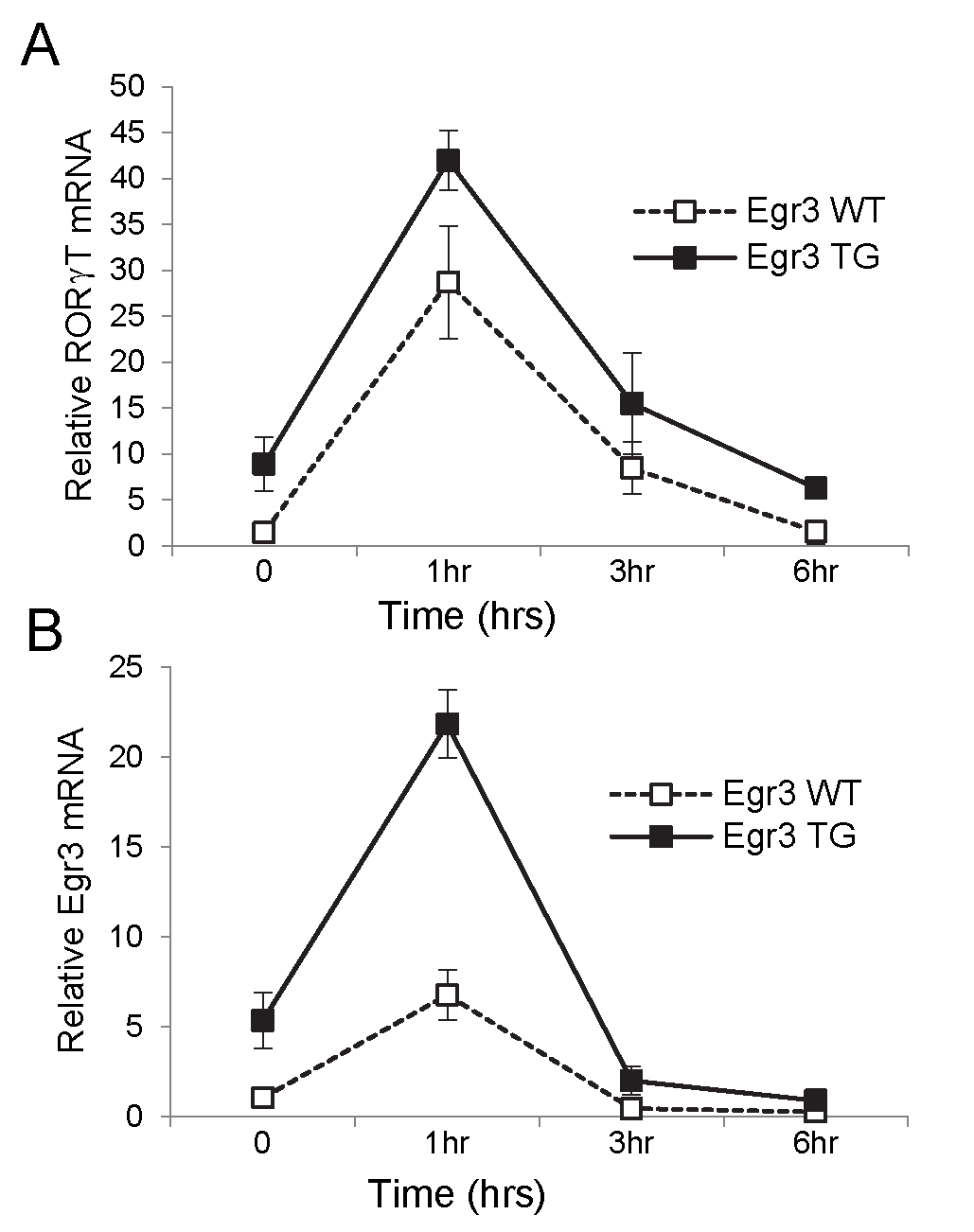

Supplement: Figure S3 — Egr3 TG T cells express higher levels of Egr3 and RORγt. Purified γδ T cells were stimulated directly ex vivo for the times specified, then RORγt (A) or Egr3 (B) mRNA expression was measured by qRT-PCR done in triplicate and normalized to 18s expression. Data is from a single experiment using cells from 3 mice per genotype. (TIFF) [file pone.0087265.s003.tiff]
